# Prolepsis and Rendering Futures in Intergovernmental Panel on Climate Change Reports

**DOI:** 10.1177/07410883231222882

**Published:** 2024-02-24

**Authors:** Ashley Rose Mehlenbacher, Sara Doody, Carolyn Eckert, Brad Mehlenbacher

**Affiliations:** 1 Department of English Language and Literature, University of Waterloo, Waterloo, ON, Canada; 2 Department of Knowledge Integration, University of Waterloo, Waterloo, ON, Canada

**Keywords:** genre studies, figures of speech, prolepsis, IPCC, climate change

## Abstract

Rhetorical figures of speech provide important analytical frames to chart how arguments operate within genres and within genre ecologies. Varieties of the figure *prolepsis* allow for the rendering of future time or fact in the present, which can be a powerful rhetorical inducement toward social and political action. In this article, we examine how anticipatory arguments drawn from complex data shape a key genre for public and policy-facing work on the climate crisis—the Intergovernmental Panel on Climate Change’s Synthesis Report’s (SYR) Statement for Policy Makers (SPM). We examine how the rhetorical figure of prolepsis operates within this genre to understand the anticipatory arguments and logics emerging from the synthesis of scientific findings and their reporting. Pairing figural studies and Rhetorical Genre Studies, we further offer an approach to investigate how these patterned operations of language might intersect in their rhetorical workings.

## Introduction

An article originally published in *Popular Mechanics* in 1912 generated attention some years ago (Popular Mechanics, 2018). “Remarkable Weather of 1911” warned of “the Effect of the Combustion of Coal on the Climate—What Scientists Predict for the Future.” Author Francis Molena explains the weather of 1911 was volatile and such extremes may be, in part, attributed to a changing climate affected by human activity. Anticipating and then rhetorically rendering the future as though it were fact, Molena writes,

It is perhaps somewhat hazardous to make conjectures for centuries yet to come, but in the light of all that is known it is reasonable to conclude that not only has the brain of man contrived machines by means of which he can travel faster than the wind, navigate the ocean depths, fly above the clouds, and do the work of a hundred, but also that indirectly by these very things, which change the constitution of the atmosphere, have his activities reached beyond the near at hand and the immediate present and modified the cosmic processes themselves. (p. 342)

Molena is deploying a figure of speech in this passage. Several figures, in fact, but we focus on one: *prolepsis* or what we will hereafter call *praemonitio*.^1^ Because of the brief horizons—relative even to our own life span—our attention affords as human beings, it is difficult not only to understand climate change on a scale of hundreds of years, extended into our past and extending into our future, but also to the future of those generations that will follow. Praemonitio moves future time into the present by demanding and redirecting our attention. Inducing rhetorical effect to generate action now that will change a distant future (relative to our experience of future time) is a powerful rhetorical operation. It is, however, also difficult to sustain. The persuasive force must generate enough motivation for action that even difficult actions are undertaken, perhaps for the remainder of one’s life.

The figure has been used in climate communications many times since Molena’s writing in 1912 and is still widely used today. Consider another example of this figure, where the anticipation includes not only climate impacts, but the likelihood that warnings given by scientists and experts will be ignored. The example is drawn from the first IPCC report, released in 1990, and concerns climate impacts on socioeconomic systems in fishery management. Explaining first the impacts—how such anticipations can be made—and then what is likely to happen when expert management strategies are ignored, the report reads:

Changes in climate will alter the species composition and productivity of marine ecosystems supporting major fisheries. As a first approximation of the ability of societies to cope with changes in abundance and distribution of marine resources, one can look at the socioeconomic impacts of such changes in the recent past. Commonly cited examples of such changes include the collapse of the Peruvian anchoveta, the Californian sardine, the Alaskan King Crab, or the expansion of such fisheries as the Bering Sea pollock and the Chilean sardine. These provide important information about how societies have dealt with major changes (favourable as well as adverse) in fishery resources availability in the recent past. For example, as fish stocks have declined because of a combination of less favourable environmental conditions and overfishing, industries have often made the problem worse by continuing heavy fishing pressure, thus accelerating the collapse of the fishery. *Frequently, industries as well as governments have been unwilling to accept the advice of scientists and take meaningful action to protect fish stocks* [emphasis added]. It is likely that global warming will produce collapses of some fisheries and expansions of others. The likelihood of collapse may be aggravated by inadequate management due to insufficient authority, unwillingness to act or lack of knowledge. (pp. 6-19–6-20)

Praemonitio makes several moves here: the first move made is a broad claim (“Changes in climate will alter the species composition . . . ”), then an analeptic move to establish grounds for these claims (“one can look at the socioeconomic impacts of such changes in the recent past . . . ”), then a move to provide evidence of such events (“collapse of the Peruvian anchoveta . . . ”), and then a move to explain the significance of this claim and these examples as grounds for anticipation (“important information about how societies have dealt with major changes . . . ”). The key move, however, follows the establishment of the proleptic vision. Adding to the vision of future impacts is a vision acknowledging that all the work of such presaging is likely to be unheeded (“industries as well as governments have been unwilling to accept the advice of scientists”). Finally, then, the vision is fully realized in the last two sentences of the paragraph (“It is likely that global warming will produce collapses of some fisheries and expansions of others. The likelihood of collapse may be aggravated by inadequate management due to insufficient authority, unwillingness to act or lack of knowledge.”).

Such examples show that praemonitio can tell us much about how we talk about climate change, the climate emergency, and climate justice. The study of figures of speech such as praemonitio can also provide new ways to investigate scientific genres (or, for that matter, any genres although we constrain our interest here to the scientific). Given the figure’s important rhetorical work, we set out to examine its use in perhaps one of the most high-profile climate change genres—the Intergovernmental Panel on Climate Change (IPCC) reports. To illustrate both the work of the figure in genres of science and how figural analysis can be deployed to understand genre, we build our analysis on studies of rhetorical figuration. Specifically, we draw on the evolving tradition of figural studies set out by Fahnestock (1999), which tell us that rhetorical figures of speech epitomize arguments, and that Harris (2013) has shown illustrate patterns of thought and reveal cognitive affinities. We are also informed by earlier work in rhetorical studies of science showing that figures function foundationally in the work of scientific thinking and writing (Halloran & Bradford, 1984). Indeed, our purpose is to illustrate the role of figures in the IPCC reports, with their complexity of success and failure and to reject the “antifigurist tradition in scientific and technical writing” that Halloran and Bradford identified 40 years ago.

Of the hundreds of rhetorical figures of speech, we selected proleptic analysis as an approach because the figure is pervasive in environmental genres. Furthermore, the proleptic analysis illustrates how figural logics and genre-ing activities work together to facilitate social action (Miller, 1984) across genre contexts. Examining the figural operations within a genre framework helps to illustrate the layered complexity of rhetorical activities. Writing of climate communication genres, Bazerman (2021) explains that the production of knowledge and knowledges are shaped by genre users, and, critically, “productions are intertextually linked to prior texts and the knowledge produced therein” (p. 36; see also: Bazerman & Rogers, 2008).

Our study focuses on one of the IPCC texts—the IPCC Synthesis Report’s (SYR’s) Summary for Policy Makers (SPM). The IPCC Summaries remain the most widely read elements of the IPCC reports by policymakers and media alike (Boykoff & Pearman, 2019) and thus have significant powers in shaping conversations surrounding climate change, action, and justice. The SPM is particularly sensitive to broader discourses (i.e., in media, policy, activism) surrounding climate change and action, and also climate justice as well as genre change. Indeed, within the context of climate change, the IPCC reports serve as a useful illustration of the interconnectedness of genres: the reports are syntheses of scholarly research, which are then used as the basis of media reporting, before influencing other genres such as the protest sign (Auken, 2021). In the SYR SPMs, the genre change is epitomized by proleptic work because as the evidence for anthropogenic climate change mounts, the SPMs gradually evolve to take on more urgency and present more vivid visions of the future, some optimistic, some less so. We suggest that this change is, at least in part, a result of how the SPMs are taken up in coverage and discussion of climate change, (see Mehlenbacher et al., 2023), with media outlets, politicians, and activists repurposing some of the anticipations presented in the summaries to make their own appeals for climate action or justice in addition to the material realities of the climate crisis becoming increasingly visible. Through this lens we can view the IPCC report as a genre that shapes and is shaped by related texts and prior iterations of the report itself.

## Review of Literature

### Praemonitio

Over the millennia, prolepsis has been used to describe different forms of anticipations, and therefore it is helpful to categorize uses into distinct subtypes. A common use of prolepsis (sometimes also called *procatalepsis*) describes anticipating an opponent’s arguments, or presumed arguments against one’s own claims, and rebutting them before anyone else can raise them. Another subtype of prolepsis functions through a kind of future anteriority, where events of the future can be known, such as in literary texts. We disambiguate these terms from the subtype of prolepsis we use here—what we will call praemonitio^2^—which refers instead to the rhetorical strategies of anticipation as forewarning. Disambiguation of distinct uses of the figure is an important step in our approach, and here relies on previous work (Mehlenbacher, 2017), but can be done for other figures by identifying uses in the so-called handbook tradition as well as by building a corpus (or collection of examples) and carefully reviewing those to mark the rhetorical work undertaken in each which can be categorized into subtypes, if present.

Understanding praemonitio’s temporal structure keys into its suasive effect. Praemonitio’s anticipatory presage is a forewarning or foretelling of a future time and future event to shape the actions of the current time by taking action (an event). Or, in other words: Event Y occurs at Time B, and Event X (action to shape Event Y) is urged to happen at Time A (see Mehlenbacher, 2023; Mehlenbacher et al., 2023). In other words, through a temporal dislocation that ruptures linear perceptions of narrative time, often relying also on analeptic features—or what we can describe as a flashback—praemonitio rhetorically renders future-time in the present. Praemonitio here forms the structure of the reasoning or argument establishing the grounds for anticipating these future visions. Although the outcomes cannot be certain, they might be reasonably anticipated. Crucially, such anticipations call the audience to action. Praemonitio is the rhetorical work of selecting key aspects from the natural, as well as social and cultural experiences, to make this potential future as realistic as possible. The key to inducing rhetorical effect is to generate an imaginable future as the near or present reality (those outcomes in present terms), to create an urgency or desire for action. Praemonitio achieves its effect through a kind of teleological prospective, which is to say, still aimed at the end or the telos, but where the “endpoint” is looked forward to gain understanding of its significance beforehand. This is essential to praemonitio in that the rhetorical strategy aims to induce some effect upon the hearer to move them to action (e.g., vote for candidates with a climate action plan).

Praemonitio is an interesting rhetorical figure of speech to examine the evolution of the IPCC reports. The reports must relate complex information that is difficult for an individual person to understand owing not merely to scientific complexity, but also to massive geographical scale and considerable temporal horizons (relative to the human life span). Figures, importantly, can work to epitomize scientific arguments (Fahnestock, 1999) and help distill complexity into memorable logical formulations. Yet, as Harris (2013) has shown, despite the compelling use of antimetabole to distill Mendel’s arguments about plant genetics, Mendel’s appeals were missed by his contemporaries studying hybridization, only to be realized later by biologists at the turn of the twentieth century. Harris is not primarily concerned, however, with the “suasive failures and deferred successes of Mendel’s epochal argument” and so we are left speculating on the reasons for these deferments (2013, p. 597). We take this case to demonstrate that, despite the deployment of rhetorical-figural strategies that elsewhere have proven effective (see Fahnestock, 1999, on Darwin’s work), such epitomizations may fail.

Praemonitio can be a highly effective rhetorical figure of speech, evocatively and urgently demanding a response. Yet its deployment, especially as related to climate change, seems to go awry at times. That is, although praemonitio is a device that has long appeared in environmental communications, the figure sometimes fails to deliver the goods—the proximal urgency or what Perelman and Olbrechts-Tyteca (1969) call *presence* (cf. Mando, 2016, on the spatially concerned “vicarious proximity”)—needed to address long-term environmental change. It remains, however, one of the more powerful rhetorical tools for rendering large-scale, temporally vast, and scientifically complex ideas into a foreseeable future vision. Each case is certain to have its contextual complexities. Our measure of importance here, in short, is derived from the actions of the scientific community itself, which has, for more than 20 years, dedicated some of the most expert researchers to compile, debate, vet, and endorse these reports.

### Figures of Speech and Genre Action

Studies of figuration in the tradition of figural logic implicate the socialization of thinking and knowledge as social negotiation through the study of rhetoric and argument, particularly in scientific communities. However, there are broader practices of communication and rhetorical negotiation that serve to shape how knowledge is produced, and studies of genre align well with studies of figuration, in particular, with what are sometimes called “figures of thought.” That is, when one studies figures of speech, there are various taxonomies attempting to distinguish figures based on their operations in language. Such operations include the syntactic configurations that mark those figures of speech called “schemes” (e.g., antimetabole; or, in terms of its grammatical construct, lexical inversion) or semantic configurations, such as departure from “literal” speech indicated in the figures of speech often called “tropes” (e.g., metaphors; consider, for instance, “truth as light”^3^). Figures of thought are sometimes a “miscellaneous” category for those figures that fit neither into schemes nor tropes. But such figures, in their complexity of function, provide an insight into an intermediate level of cognitive-textual-social recurrences between figures as schemes and tropes and rhetorical genres.^4^ Prior to making this case further, it is useful to briefly examine the nature of rhetorical genres as a theoretical construct.

Key to our definition of genre is that of *uptake* (Freadman, 1994, 2002), the ways in which genres are responded to and interact within social situations, or the call-and-response cycle of an intertextual, communicative chain (cf. Bakhtin, 1986). During genre uptake, genres themselves are modified as they are subjected to a “feedback loop” (Freadman, 2020, p. 112) of social expectations. Significantly, the ways in which genres are, or are not, taken up allows us to see how genres “coordinate complex forms of social action” and, significantly, “how and why genres get taken up in certain ways and not others, what gets done and not done as a result” (Bawarshi & Reiff, 2010, p. 86). Uptake, in other words, is a way in which earlier genres are recognized by later responses in the communicative chain that “confirms the status [of a preceding genre] by taking the information therein as worthy of repeating or re-representing” (Cooke, 2021, p. 178). In the case of climate change, the IPCC report, which is taken up by a variety of stakeholders (e.g., media, policymakers, activists), each uptake has the potential to trigger an evolutionary response in the genre. Note, too, that it is often through responses that information in antecedent genres (Jamieson, 1975) is marked as being especially worthy of reporting and representation.

Traces of these uptakes can be charted through the figural logics that move arguments from one genre into another. While some arguments may transform in the sense described by Fahnestock (1986) as accommodations, or the recasting of scientific knowledge suited to a public audience’s needs, the argument logics marked by figures of speech can help us follow where scientific knowledge moves unaccommodated and accommodated into other genres, including public-facing science communications.

Praemonitio is a logic rendered in IPCC reports, as we will argue in the following analysis, and it is also found in media coverage of these reports (Mehlenbacher et al., 2023). Although the IPCC reports are not a research process genre (Swales, 1990) and could perhaps be characterized as a parascientific genre (Mehlenbacher & Miller, 2018) wherein the research process genres underlying scientific research on climate change are taken up in the reports and provide a site themselves for public communication or media uptake. As such, the IPCC reports are an important player in the genre ecologies contributing to scientific understanding and knowledge as much as climate action. To better understand how the IPCC report genre functions and how figural analysis might help us trace arguments as they move through entailed genre ecologies, we now turn to our analysis of the IPCC synthesis reports.

## The Intergovernmental Panel on Climate Change

The Intergovernmental Panel on Climate Change (IPCC) is an organization established in 1988 with the mandate to provide policy makers with up-to-date knowledge regarding the science of climate change. The First IPCC Assessment Report (FAR; or Assessment Report 1 [AR1]) was published in 1990, the Second Assessment Report (SAR; or AR2) was published in 1995, the Third Assessment Report (TAR; or AR3) was published in 2001, the Fourth Assessment Report (AR4) was published in 2007, the Fifth Assessment Report (AR5) appeared in 2013 to 2014, and the Sixth Assessment Report (AR6) was completed in 2023.

The reports are, in fact, composed of multiple reports drafted by three different working groups (WGs). The WGs compose reports on different aspects of climate science and, following the completion of those reports, a Synthesis Report is drafted. The final Assessment Report, thus, includes four texts: Working Group I (WGI), Working Group II (WGII), Working Group III (WGIII), and Synthesis Report (SYR). Each report has, normally as its first chapter, a Summary for Policy Makers (SPM), which is the most widely read and reported on element of the reports (Boykoff & Pearman, 2019).

In addition to SYR’s reference to the WG reports, as well as other special reports issued in the interim between ARs, previous reports are recalled. For instance, the policy summary in the AR6 references the AR5 (e.g., in reference to sensitivity of models on p. SPM-13, also in comparing predictions like on pp. 10-103) and also calls back to the AR4 (e.g., when warning of the climate described as “unequivocal” on p. TS-8; see also pp. 1-5, 1-45). These references report that, per the findings, climate change was unequivocally happening in 2007, that human cause was extremely likely in AR5, to saying climate change is happening and humans are unequivocally causing it in the AR6.

Beyond the complex drafting process, the IPCC assessment reports are products of a complex review process involving an international community of experts on various aspects of climate science. The reviews involve an enormous number of experts with the first reviews including scientific experts and working groups reviewing for accuracy, the second review involving policymakers, and the third review engaging governments, culminating in some 142,631 comments overall.^5^

Given that the IPCC report serves complex audiences and social actions, and addresses a global threat that involves aspects of climate but also entails matters of health, social wellbeing and justice, security, and more, the genre-ing activities of the IPCC reports occur in an unfolding situation. Here multiple audiences must be addressed and numerous strategies are at work to do so. For example, in the AR2 Synthesis Report, the Foreword explains that the Summary for Policymaker section must “reflect the state-of-the-art understanding of the subject matter,” and, of interest to our questions of audience, “be written in a manner that is readily comprehensible to the non-specialist” (IPCC, 1995, p. vii). The next sentence, however, indicates a significant challenge in reporting technically state-of-the-art science while providing nuance, adding, “Differing but scientifically or technically well-founded views should be so exposed in the reports and the SPMs, if they cannot be reconciled in the course of the assessment” (IPCC, 1995, p. vii). The AR4 similarly notes that for the SYR, “the authors prepared the draft in a non-technical style while ensuring that scientific and technical facts are recorded correctly” (IPCC, 2007, p. v). The AR5 explains, in the SYR, that “to facilitate access to the findings of the SYR for a wide readership and to enhance their usability for stakeholders, each section of the SPM carries highlighted headline statements” (IPCC, 2014, p. vii).

Finally, because the IPCC report is a genre aiming to address the urgency of climate change, it is notable how communicating this urgency has evolved over iterations of the report. The AR6 is more explicit about the urgency of climate change than previous iterations of the genre. One example of this can be traced in Working Group II (Impacts, Adaptation, and Mitigation) reports. Because Working Group II is concerned with climate impact, there is naturally a focus on the urgency of climate change and its effects on human life, yet we observe that the AR6 begins, in Chapter 1, with a discussion entitled “The Current Urgent Moment,” language that is not used to frame the AR5 report. In fact, the AR6 goes to some effort to emphasize how the urgency of climate change has increased since the AR5 was released: it observes that more of the global population views climate change as an emergency than ever before, mentions of climate change in global media have increased, and notes that business communities now increasingly view climate change (and climate failures) as major risks (IPCC, 2022, pp. 1-8). We also observe that urgency is communicated through climate mitigation and adaptation, with the AR6 emphasizing the importance—but also the real possibility—of adopting policies to curb warming and adapt to climate events that have already been triggered.

IPCC reports in some ways echo Miller’s (1980) early findings on Environmental Impact Statements (EIS) in the United States. A largely unsuccessful genre at the time, which she describes as “troubled from the start” (Miller, 2015, p. 57), the EIS faced numerous challenges in determining its audience and purpose, with its broad intended audience presenting a significant challenge to the genre because of differing audience needs. Bazerman (2021) notes that the US Environmental Protection Agency published a report in 1983 warning of the impacts of climate change, with “serious consequences for food production and sea levels,” and there were also several UN-sponsored panels that preceded the first IPCC assessment report (p. 40). Critically, Bazerman also notes how uncommon it is in scientific knowledge production to see the formation of such panels, rather than reliance upon traditional mores of review. However, we can understand the function of these panels and reports, Bazerman explains, by understanding that for government and intergovernmental policy and action, such authentication and sanctioning is important, alongside “belief and commitment of segments of the population and institutional groups who will have to cooperate with the action” (2021, p. 41). Furthermore, action is generated through democratic deliberation and engagement, and messaging must not simply end up in the hands of prospective policymakers but their constituents. In this way, he reports serve important functions beyond their originating genred form. Media uptake of IPCC messaging, for instance, serves to share the calls for action on the climate crisis. One approach to tracking such uptakes could be through a figural analysis of messaging. Indeed, in previous research (Mehlenbacher et al., 2023), we found prolepsis in media coverage of IPCC reports. Here we examine the IPCC reports in detail using figural analysis, with particular attention to how prolepsis highlights the urgency of what can often seem to be an emergency of a distant future time.

### Methods

Recurring and typified social actions consist of meaningful, and meaning-making, rhetorical *moves*, or “discoursal or rhetorical [units that perform] a coherent communicative function” (Swales, 2004, p. 228) within a genre. Such moves are composed of textual patterns, lexico-grammatical features, and rhetorical figures that serve certain functions within, and signal recognition of, the genre itself. It might seem sensible, especially when examining a report genre, to examine what moves are made to rhetorically characterize these social actions. While we believe this is a useful approach, it is not the one we take. Rather, we look to prolepsis to help illuminate the rhetorical ecosystem within which the social actions of these reports emerge, evolve, and effect change. Put another way, while a rhetorical move analysis would buy us an understanding of how the report’s structure shapes the social or rhetorical actions the report aims to take, an analysis of prolepsis buys us a more holistic view of how those rhetorical actions may move through other genres—for instance, prolepsis may be used to mark moves meant to encourage climate action or risk mitigation. Exploring how such moves—as well as the figurations that mark them—evolve over time allows us to trace how the IPCC report itself evolves in response to how it is, or is not, taken up in the chain of textual and knowledge production.

To investigate how the use of prolepsis may be deployed to signal a renewed urgency toward climate change in the most recent IPCC assessment report requires examining several IPCC reports. The IPCC reports themselves consist of assessments of three Working Groups, focusing on different aspects of climate change, and an SYR that synthesizes the information detailed in the Working Group assessments. We began by collecting published reports in their entirety, generating a corpus of 20 total reports, 6 of which are the focus of our figural analysis (i.e., each of the 6 SYRs). Figural analysis of praemonitio was conducted on a specific section of the SYR, the Summary for Policymakers (SPM).

Genre analysis began by indexing the overall organizational features of the reports that contribute to its typification, which include navigational apparatus, including table of contents and indexes as well as informational organization such as sections, subheadings, etc., and tables, figures, images, graphics, etc. One of the most notable differences is the increased length of reports over time. For instance, the physical science working group’s report was 414 pages in 1990, stabilizing at around 1,000 words between 2000 and 2007, rising to 1,552 pages in 2013, and later, more than doubling to 3,949 pages in 2021—perhaps unsurprising given the general increase in scientific research and outputs (Bornmann et al., 2021; Wong, 2019). This increase in the length of the reports also represents increased multidisciplinarity of the authors and represented work, as well as expanded inclusion of relevant knowledges, such as Indigenous knowledges (Ford et al., 2016) and practitioner-based evidence (Viner & Howarth, 2014).

Given our interest in understanding both how praemonitio is deployed through genre-ing activities in the IPCC reports and how this figure is implicated (successfully or otherwise) in the action of policymakers on climate change as a whole rather than as its scientific, impactive, or mitigative parts, we focused our analysis of genre and prolepsis on the SPM sections (a total of 150 pages) in the SYRs (see Table 1). As mentioned, the SPMs are generally the most widely read and reported on portions of the reports (Boykoff & Pearman, 2019), particularly given that these are written specifically for policymakers, and those included in the SYR are particularly pertinent here as they synthesize the major findings and recommendations of all three working group reports. Within these SPMs, we conducted an analysis of the figure of praemonitio combined with a rhetorical move analysis (Swales, 2004) to determine whether and how the figure of praemonitio contributes to the rhetorical work of the genre of the IPCC reports and consequently to larger, normalized patterns of climate communication. The move analysis consisted of iterative readings of SYR SPMs to generate a preliminary rhetorical move structure of the report, which was informed by discourse features like headings and subheadings, the content of the reports, and images and graphs accompanying the text (Tardy & Swales, 2016). Subsequently, rather than tracing linguistic or syntactical patterns, the analysis focused on the content of each move with particular attention to how prolepsis worked to structure the rhetorical function of each move. A condensed sample of this analytic process is outlined in Table 2.

**Table 1.** IPCC Reports and Contents. Bolded Contents Indicate the Focus of Analysis.

Report Name	Report Contents (Working Groups and Summaries)
First Assessment Report (FAR)	Working Group I (WGI), Working Group II (WGII), Working Group III (WGIII), **Synthesis Report (SYR), Summary for Policy Makers (SPM)**
Second Assessment Report (SAR)	Working Group I (WGI), Working Group II (WGII), Working Group III (WGIII), **Synthesis Report (SYR), Summary for Policy Makers (SPM)**
Third Assessment Report (TAR)	Working Group I (WGI), Working Group II (WGII), Working Group III (WGIII), **Synthesis Report (SYR), Summary for Policy Makers (SPM)**
Fourth Assessment Report (AR4)	Working Group I (WGI), Working Group II (WGII), Working Group III (WGIII), **Synthesis Report (SYR), Summary for Policy Makers (SPM)**
Fifth Assessment Report (AR5)	Working Group I (WGI), Working Group II (WGII), Working Group III (WGIII), **Synthesis Report (SYR), Summary for Policy Makers (SPM)**
Sixth Assessment Report (AR6)	Working Group I (WGI), Working Group II (WGII), Working Group III (WGIII), **Synthesis Report (SYR), Summary for Policy Makers (SPM)**

**Table 2.** Sample Analysis for Move A.

SYR-SPM Move	Discourse Features and Proleptic Markers
Move A: summarizing observed changes in climate and their current effects	Headings: “Observed Changes in Climate and their Effects” (IPCC, 2007, p. 2) Content: Outlining observed climate changes often drawing heavily on Working Group I, temporal graphs tracing climate change over time, charts visualizing increase in CO_2_ emissions over time. Proleptic Markers: Largely analepsis, or flashbacks (“Over the period 1992 to 2011, the Greenland and Antarctic ice sheets have been losing mass (high confidence), likely at a larger rate over 2002-2011” [IPCC, 2014, p. 4])

## Figuring Rhetorical Moves in the Synthesis Report Summary for Policymakers

Broadly, the rhetorical situation for the IPCC reports has been shaped by scientific discourses, especially in the natural sciences, and the norms of scientific communication practices. What knowledge counts in these reports has shaped the typifications we found in the reports, but, critically, also what knowledge itself is counted.

Notably, various forms of knowledge have not been included, creating a “representation dilemma, such as local knowledges or more interpretive knowledge” (Dudman & de Wit, 2021, p. 2). How knowledge is reported to, further scientific norms and values is a negotiation of scientists with politicians. For instance, climate scientist Michael Mann explains how the development of the 1995 (AR2) report gathered enough scientific evidence to indicate that climate change was indeed primarily responsible for warming trends. During the process, a debate around phrasing occurred between scientists and government delegates of “major oil-exporting nations,” specifically over the word “appreciable” being used with respect to human influence (Mann, 2021, pp. 28, 29). The word and debate surrounding it was the subject of deliberations for two days in the drafting of the Summary for Policymakers, Mann recounts, with “discernible” winning out (Mann, 2021, p. 29). Rhetorically, the difference in “appreciable” influence of humans and “discernible” influence is significant, but the process’s rhetorical nuances are not limited to the words characterizing the changes. Mann also explains that, when the UN Climate Change Conference in Poland was held in 2018, the findings of the meeting made it clear that climate change is happening, caused by humans, and, critically, carbon reduction is an immediate action that needs to be taken at scale. Once again “petrostates,” Mann explains, “refused to support a motion to ‘embrace’ the findings of the new report” and rather agreed to “note” them (Mann, 2021, p. 105).

From Mann’s account we note that, first, specificity beyond argument move structure (i.e., word choices, for instance) is crucially important to social action. Second, we note how urgency is communicated in the SYR Summary for Policymakers (SPM) and believe this marks those synthesis reports as a critical site of study. Within these SPMs, we observe that climate urgency is communicated using praemonitio and that in IPCC reports the figure often presaged possible climate futures.

### Praemonitio’s Role in Communicating Urgency

Across all iterations of the SYR, praemonitio appeared to serve an important figural role in the communication of climate urgency, particularly in discussions of impacts and response strategies. We see such a pattern in the First Assessment Report (FAR) where climate impact projections are forecast, as through the following example, “Working Group I has also predicted the increase in global mean temperatures to be about 1°C above the present value by 2025 and 3°C before the end of the next century” (IPCC, 1992, p. 53). The same forecasting can be seen in the SYR SPM of the Second Assessment Report (SAR) in the description of a potential emissions scenario: “In all cases, the atmospheric concentrations of greenhouse gases and total radiative forcing continue to increase throughout the simulation period of 1990 to 2100” (IPCC, 1995, p. 5).

Throughout the publication of the IPCC reports, we traced how praemonitio has been deployed to highlight the urgency of climate action, but also the consequences we might expect to see if action is or is not taken. Consider, for instance, the following statement from the AR6 SYR SPM:

Continued greenhouse gas emissions will lead to increasing global warming, with the best estimate of reaching 1.5°C in the near term in considered scenarios and modelled pathways. Every increment of global warming will intensify multiple and concurrent hazards (high confidence). Deep, rapid, and sustained reductions in greenhouse gas emissions would lead to a discernible slowdown in global warming within around two decades, and also to discernible changes in atmospheric composition within a few years (high confidence). (IPCC, 2023, p. 12)

In this statement, we see a claim about rising temperatures, which is followed by—and indeed enhances—the urgency of taking actions to dramatically decrease CO_2_ and other emissions soon. Such claims work to bring potential future disaster into a more present temporal frame: things have the potential to become bad and unless we act now, this future will likely come to pass; that is, Event Y (temperatures reaching 1.5°C) at Time B (in the near term), and Event X (deep, rapid reductions in CO_2_) is urged to happen at Time A (coming decades).

### Figuring Praemonitio in the SYR SPMs

It appears the IPCC has long relied on praemonitio to communicate the urgency of climate change and climate change actions, but we might look to more recent iterations of the report and, in particular, the SYR SPMs to trace how the use of the figure has evolved over time and to what end. Given that SYRs highlight the most crucial developments from working group reports for policymakers and those who wish to take action, it is perhaps unsurprising that praemonitio features heavily in the SPMs of these reports, especially when discussing future risks and mitigation strategies. In all versions of the SYR, SPMs tend to be written using the following moves:

a) a summary of observed changes in climate and the effects these changes are currently having,
b) specific ways that observed and projected climate changes will impact various sectors (e.g., agriculture, water ways, human health),
c) suggestions of adaptation and mitigation strategies, including the economic and social dynamics of such strategies, and
d) longer-term, future projections about climate change and climate action.

Within this broader genre “move structure” – to borrow Swales’s (1990) terminology – anticipations in the form of praemonitio served an important suasive function through its predictive work. Except for Move A, which tends to use flashbacks (i.e., *analepsis*; see Mehlenbacher, 2017, 2023) rather than flashforwards to emphasize what we know now about the science of climate change, anticipations play an important role in both how the SYR SPM is realized rhetorically and in how it evolves over time to mark urgency in climate change discourse. Analepsis is critical to the generation of a proleptic logic in science communications because to render a future time one must draw on past evidence and knowledge. Moreover, it is not merely rote recall but rather a kind of recollection (cf. Carruthers, 1990) or reconstruction of past knowledge (Brescó de Luna, 2017) in a meaningful manner. That is, in the case of the IPCC reports broadly, and the SYR SPM specifically, the work to understand historical trends, data, and make sense of the growing body of scientific literature involves extensive understanding of the existing record of research. Indeed, as these reports synthesize the enormous and growing literature on climate change, much of the work is looking back such that we might have an informed understanding of what is to come. In this way we see that praemonitio is no mere dressing on the work of scientific thinking, nor is it just the important work of collective memory (Brescó de Luna, 2017), but is also a foundational figural logic that aids in the creation of scientific knowledge. Much of the knowledge in the SYR SPM is that which is to be mobilized, or put to work in the service of society, and in this way the figure of praemonitio becomes essential to epitomizing the argument of these reports: climate change is happening (past), we humans are mostly responsible and making it worse every day (present), and it is going to significantly impact how we live (future).

Anticipations can be traced in discussions on climate impacts where possible climate futures are emphasized, but solutions are not offered. The FAR SYR SPM for instance offers one vision of the future where we see “an effective doubling of CO_2_ in the atmosphere between now [1990] and 2025 to 2050” (IPCC, 1992, p. 53). Likewise, the AR4 SYR SPM in its discussion of impacts predicts “GHG [greenhouse gas] emissions at or above current rates would cause further warming and induce many changes in the global climate system during the 21^st^ century that would *very likely* be larger than those observed during the 20^th^ century” (IPCC, 2007, p. 7). Both anticipations offer visions of the future, but not actions to prevent such futures from coming to pass; in other words, while these visions show us potential futures, they do not explicitly endorse or call for action, a key element for effective rhetorical use of praemonitio. We do see such calls to action in sections of the report outlining adaptation and mitigation strategies; for instance, in a section titled “Technology and Policy Options for Mitigation” the SAR SYR SPM notes that “significant reductions in net greenhouse gas emissions are technically possible. . . . These reductions can be achieved by utilizing an extensive array of technologies and policy measures” (IPCC, 1995, p. 12). Here, we are presented with a possible future with only vague suggestions about how to get there. In the AR5 SYR SPM, we again are presented with a specific vision of a future in which climate change may be mitigated and some actions that might enable this future to come to pass:

Substantial emissions reductions over the next few decades can reduce climate risks in the 21^st^ century and beyond, increase prospects for effective adaptation, reduce the costs and challenges of mitigation in the longer term, and contribute to climate-resilient pathways for sustainable development. (IPCC, 2014, p. 17)

While we are provided with actions that will mitigate climate change and help ensure a more sustainable future, the anticipatory vision does not inspire a great sense of urgency: the social actions we are meant to be persuaded into performing lack the crisis context that we observe in Move B.

Perhaps the most suasive instances of praemonitio realize anticipations in Move D, where the SYR SPMs report on questions to be explored in future IPCC reports and where long-term perspectives are considered (note, these long-term perspectives may at times include further discussions about mitigation and adaptation but are more concerned with potential uses). In these sections, particularly in more recent iterations of the report (e.g., from the AR4 onwards), proleptic figures realize the urgency of climate change and use this sense of urgency to (hopefully) induce readers to action. Consider an example from the AR4, where the urgency underlying proleptic calls to action becomes more pronounced. In its “Future Directions” section, the report simultaneously warns and urges readers to action:

Many impacts can be reduced, delayed or avoided by mitigation. Mitigation efforts and investments over the next two to three decades will have a large impact on opportunities to achieve lower stabilisation levels. Delayed emission reductions significantly constrain the opportunities to achieve lower stabilisation levels and increase the risk of more severe climate change impacts. (IPCC, 2007, p. 19)

We are urged, “over the next two to three decades” (Time A), to make efforts to mitigate emissions and invest in sustainable technologies (Event X) so that we can stabilize emissions (Event Y) in the future (Time B). Simultaneously, readers are warned that *inaction* (Event X) now (Time A) will make it much harder to control emissions and will make more severe climate impacts more likely (Event Y) in the future (Time B). Here, praemonitio is employed to cleverly play with time by creating two possible futures and forcing us to decide which one to pursue.

The AR5, too, uses praemonitio as a figure to mark urgency and call for action in its final section exploring longer-term consequences of mitigation and adaptation. Interestingly, such urgency is more often located in the explanations of mitigation and adaptation strategies that will be most beneficial in the long term, rather than in the report headings. Consider the following example that overtly emphasizes the timeliness of action:

Aligning climate policy with sustainable development requires attention to both adaptation and mitigation (*high confidence*). Delaying global mitigation actions may reduce options for climate-resilient pathways and adaptation in the future. Opportunities to take advantage of positive synergies between adaptation and mitigation may decrease with time, particularly if limits to adaptation are exceeded. (IPCC, 2014, p. 31)

Here the figure advises us on the actions to take (Event X; aligning policy and development) *now* (Time A), before we lose opportunities (another form of Event Y; limited pathways) to do so in the future (Time B). The temporal rendering this figure offers is partially dependent upon a sense of kairos. Kairos, as embedded in this figure, makes clear that unless action is taken soon—the opportunity seized—there may be no opportunity for action in the future—acting on climate change has yet to become *akairotic*. This is especially notable given that each iteration of the report has increasingly attended to the urgency of action: there is still time to act, but the window for meaningful action is growing steadily narrower. Praemonitio, then, induces social responses to climate change while they can still make a meaningful and positive difference for the future. Indeed, more recent iterations of the SYR SPM include an increasing presence of illustrated figures in the report that appear to invoke praemonitio to induce action. An especially illustrative example shows, in a figure, both analeptic visions of how inaction on climate change through “missed opportunities” have led to the current urgent moment, as well as multiple possible futures that might be achieved by acting in a “rapidly narrowing window . . . to enable climate resilient development” (IPCC, 2023, p. 29). Such illustrations are suggestive of how rhetorical figures like praemonitio might be deployed visually, in addition to textually, and offers a potential avenue for future exploration of this figure (see the figure titled “Figure SPM.6” in IPCC, 2023, p. 29).

We might also look to the coverage of the Working Group I SPM to see more recent examples, where urgent appeals to action were accompanied by proleptic visions of possible climate futures. Some changes were reported in both the SPM and media coverage as being “locked in” or set to happen (e.g., the Arctic seeing a sea-ice free summer by 2050) but were accompanied by appeals to action. Indeed, one of the final points from this SPM states that “if global net negative CO_2_ emissions were to be achieved and be sustained, the global CO_2_-induced surface temperature increase would be gradually reversed but other climate changes would continue in their current direction for decades to millennia” (IPCC, 2021, p. SPM-39). In our proleptic structure, we might break the example down as follows: Event Y (both gradual reversal and “other climate changes”) at Time B (decades to millennia), and Event X (“global net negative CO_2_ emissions”) is urged to happen at Time A (implicit via achieved/sustained). Such visions, which both emphasize the urgency of our current time, while providing a more optimistic vision for the future, were taken up in public discussions of climate change and shaped the tone of the discussion. In media coverage^6^ of the AR6 specifically, such visions were taken up and further disseminated in outlets like *The New York Times* (“humanity can still prevent the planet from getting even hotter . . . which would entail a rapid shift away from fossil fuels starting immediately” [Plumer & Fountain, 2021, para. 7]), illustrating how the SPMs are instrumental in shaping discourses surrounding climate change, but also in how broader discourses are instrumental in shaping climate action.

## Concluding Remarks and Considerations

Within the IPCC Synthesis Reports’ Summary for Policymakers, the rhetorical figure of praemonitio works to structure and facilitate the critical social action for which the genre calls. The urgency that praemonitio is meant to communicate sometimes does so at the expense of hope: while the warnings in the IPCC reports are indeed effective in catching the attention of policymakers, media, and publics, their function is not necessarily to offer avenues for optimistic action, which can undermine the use of praemonitio as a rhetorical figure. Providing pathways for everyday people to get involved in climate action and working toward climate justice is an important element to consider.

Predicated upon this suasive appeal is that one believes there is a temporal horizon, a future, within which they might act to influence, thus entailing ethical decision-making. Where praemonitio’s ethical work is most evident is in the rhetorical actions it induces. Although the ethical entailments of praemonitio are not only these pragmatic effects, we are also interested in these because they are important to the suasive strategies used in the reports we study here. Praemonitio is based upon not merely an anticipation but rhetorical work that serves to shape a vision of possible futures and possible social action. In the case of climate change, when a rhetor presages a possible and likely future, they must make choices about how to represent that future as well as the present possibilities to affect or change this presage. Effecting change is central to the figure’s strategy and to its success. In environmental discourses, praemonitio is often balanced between imagining a future we fear, and the hope needed to change that future. Where a praemonitio leans into fear, it may have negative consequences to the social action that the appeal attempts to induce through persuasive affect, such as generating extreme eco-anxiety that results only in fear of the future, hopelessness, or inaction. This tension is an ethical problem in the formulation of praemonitio as far as the rhetorical affect it induces operates against a social, civic response to an impending or ongoing crisis.

Recalling trends that move the climate change debate outside of scientific arguments, there is a danger for the use of praemonitio to epitomize scientific arguments, one that should be considered in crafting of a particular use of the figure. Because one of the strategies of climate skeptics is not to deny scientific evidence itself but to suggest that reactions to the science and the warnings it entails are disproportionate to the actual risk, use of praemonitio must itself anticipate such co-opting.^7^ That is, employing praemonitio, while effective at epitomizing arguments, also distills key arguments that can then be used by bad faith actors or in the spread of conspiracy myths.^8^ This is a critical concern when we look to understand the rhetorical potential and power of a figure such as praemonitio because the rendering of future time or fact in the present can be a broadly powerful rhetorical inducement toward social and political action as well as inaction.

Although understanding climate change relies significantly on scientific work, now is a critical *rhetorical* moment for climate action and climate justice as decisions now lie in the hands of policymakers. One climate scientist, Sonia Seneviratne, who participated as a coauthor on three IPCC reports, strongly framed the problem, writing that “We have all the evidence we need to show we are in a climate crisis” (Thomson Reuters, 2021, “UN sounds alarm on ‘irreversible climate impacts, but offers hope”). Seneviratne then frames the utility of issuing more IPCC reports, saying “Policy makers have enough information. You can ask: Is it a meaningful use of scientists’ time, if nothing is being done?” (Thomson Reuters, 2021, “UN sounds alarm on ‘irreversible climate impacts, but offers hope”). Indeed, rhetoric has much work to do to aid in understanding why appeals to climate action may be unsuccessful. As we presage what climate impacts will continue to unfold in the future through narrative, predictions that were decades if not a century or more in the making (by scientists, Indigenous leaders and communities, and environmental activists), become the present as the once-anticipated future arrives.

## Notes

1. Wait, you might say, prolepsis describes the anticipation and rebuttal of an argument—and you would not be wrong. However, prolepsis describes multiple figures of speech throughout the millennia of its deployment to describe rhetorical strategies of anticipation. As a rhetorical concept, prolepsis broadly affords the anticipation of some future time. We explain figural subtyping in the Methods section, and more detailed subtyping can be found in Mehlenbacher (2017, 2023).

2. We, therefore, for the sake of clarity, proceed with the use of “praemonitio” as the key term for this figural device, but where we refer to “prolepsis” or “proleptic” stances, etc., it should be understood we also mean this variety.

3. On this metaphor, and different theorizing of this figure of speech and others, see Mehlenbacher (forthcoming).

4. We might call these moves, as Harris (2013) has and with distinction from tradition of Swalesian moves in genre studies. Swales (1990) moves are instructive, as we will discuss, but they are distinct from figural moves or what Fahnestock (2004) has called “pragmatic.”

5. IPCC Secretariat. (2015). IPCC Factsheet: How does the IPCC review process work? Available online: https://www.ipcc.ch/site/assets/uploads/2018/02/FS_review_process.pdf

6. Earlier work suggested that ethical practices inherent to journalism, such as balanced reporting, may have inadvertently created a false perception of debate. More recent studies suggest coverage of climate change science in the popular press is largely accurate today. McAllister et al. (2021) report an analysis of 4,856 articles from January 2005 to December 2019 across five countries and found that balanced reporting “has not informationally biased coverage of anthropogenic climate change from 2005 to 2019” and that “on average scientifically accurate climate coverage improved,” although right-leaning outlets tend to be less accurate (p. 11).

7. Argument dynamics of the “climate debate” have been long documented as a manufactured controversy (Ceccarelli, 2011; see also, Leiserowitz et al., 2013). Further, Bazerman (2021) details how industry and organizations have interfered with knowledge about climate change impacts, including rhetorical efforts to frame the issue around uncertainty. Smart and Falconer (2021) examine how climate information is not only decontextualized but recontextualized for denialist arguments. Consider “climate realist” narratives that frame those urging action as “alarmists” and researchers have found that these terms and their cognates saw a 900% increase (from January 2016 to March 2020) in use on Twitter, noting a significant increase in 2018 to 2019 (Levantesi & Corsi, 2020, para. 4).

8. Corsi (2021) examined 4chan’s /pol/ discussion board and used topic modeling to identify three key clusters around climate debates, one of which concerned science, another politics, and the third hate speech and conspiracy myths (“Results,” para. 1). In the latter cluster, five topics were identified: “*race*,” “*antisemitism*,” “*climate nationalism*,” “*offensive language*,” and “*conspiracy theories*” (“Results,” para. 7). Such “indirect” climate skepticism points, as Corsi describes them, are notable for their increase, with, for instance, racist discussions increasing by almost 31% and antisemitic comments increasing by 40% between 2015 and 2019 (“Results,” para. 12).
